# Swallowing Evaluation in Patients With Unilateral Vocal Fold Immobility

**DOI:** 10.4021/gr270w

**Published:** 2010-11-20

**Authors:** Geruza Costa Gonzaga Aneas, Hilton Marcos Alves Ricz, Francisco Verissimo Mello-Filho, Roberto Oliveira Dantas

**Affiliations:** a Department of Medicine, Medical School of Ribeirao Preto, University of Sao Paulo, Ribeirao Preto, SP, Brazil; b Department of Ophthalmology, Otolaryngology / Head and Neck Surgery Medical School of Ribeirao Preto, University of Sao Paulo, Ribeirao Preto, SP, Brazil

**Keywords:** Pharynx, Swallowing, Upper esophageal sphincter, Vocal fold immobility, Deglutition

## Abstract

**Background:**

Unilateral vocal fold immobility is the neurological disorder most frequently seen in the larynx that may cause swallowing dysfunction. The objective of this investigation was to evaluate the oral and pharyngeal phases of swallowing in patients with unilateral vocal fold immobility.

**Methods:**

It was evaluated by videofluoroscopy of the swallowing of 14 patients with unilateral vocal fold immobility and 11 control subjects. The examination was performed with swallows of 5 mL and 10 mL of liquid and paste boluses. The oral transit, pharyngeal transit and clearance, the duration of upper esophageal sphincter (UES) transit, the duration of the hyoid movement, and the timing of the events were measured.

**Results:**

With swallows of 10 mL of liquid bolus (controls: 0.23 ± 0.04s, patients: 0.27 ± 0.05s, p = 0.03) and 5 mL of paste bolus (controls: 0.18 ± 0.04s, patients: 0.22 ± 0.04s, p = 0.01) there was a longer duration of UES transit in patients compared with controls. The UES opened earlier in the control subjects with the increase in bolus volume from 5 mL to 10 mL (p < 0.05), an effect that was not seen in patients with vocal fold immobility.

**Conclusions:**

We conclude that patients with unilateral vocal fold immobility may have alteration of bolus transit through the UES and have no adaptation in the swallowing timing related to the increase in bolus volume.

## Introduction

Unilateral vocal fold immobility is the neurological disorder most frequently seen in the larynx [1, 2]. Frequently, it is the consequence of lesion of the innervation [3], resulting in voice alteration as the most important manifestation.

The immobility may be caused by neck and thoracic surgery, which can impair the innervation of the pharynx, upper esophageal sphincter (UES), and proximal esophagus, but may also be due to malignancy, trauma, intracranial causes, or may be idiopathic [4, 5].

Besides the voice alteration, the lesion that causes vocal fold immobility should also affect the pharyngeal phase of swallowing. Abnormalities of supraglottic laryngeal and pharyngeal functions were found in patients with recurrent laryngeal nerve injury and ipsilateral vocal fold immobility [6, 7]. An increase in pharyngeal contraction amplitude and reduced pharyngoesophageal wave durations were reported in patients with vocal fold motion impairment [7]. If the paresis of the recurrent laryngeal nerve is associated with paresis of the superior laryngeal nerve, there is impairment of the pharyngeal constrictor musculature [6]. Patients with unilateral vocal fold immobility have laryngeal penetration and aspiration with a thin liquid bolus, but it is not frequent with a paste bolus [1, 4].

Our aim in this investigation was to evaluate by videofluoroscopy the oral and pharyngeal phases of swallowing in patients with unilateral vocal fold immobility. Our hypothesis is that the lesion that causes vocal fold immobility can also affect the pharyngeal function during swallowing.

## Materials and Methods

It was studied the swallowing of 14 patients with unilateral vocal fold immobility and 11 control subjects. The patients were 3 men and 11 women aged 30 - 72 years, median 61 years. All had voice alteration and videolaryngoscopic examinations showed immobility of the vocal folds on the left side in ten and on the right side in four. In nine cases the immobility was seen after tyroidectomy, in two after a diagnosis of lung cancer, and in three it was idiopathic. The patients did not have heartburn, acid regurgitation, other neurologic disease, stroke, hypothyroidism, or dyspnea. During the meals, 12 patients reported mild dysphagia, 10 patients had cough, and 4 had fatigue. All patients were subjected to electromyographic (EMG) evaluation of the cricothyroid and thyroarytenoid muscles in the Department of Neurology of the University Hospital of Ribeirao Preto. The examination and analysis were performed as previously described [8]. In the normal cricothyroid muscle the electrical output increases greatly when a high-pitched noise is performed, and in the normal thyroarytenoid muscle the electrical activity increases during glottal stop (valsalva), increases with inspiration and decreases with expiration [8]. Five patients had EMG alteration in the thyroarytenoid muscles, three in both muscles, and six had no EMG alteration. The time between voice alteration consequent to vocal fold immobility and the swallowing evaluation ranged from 11 days to 240 months, median 11 months, with a median time of 10 months (11 days to 40 months) in patients with EMG alteration and 23 months (40 days to 240 months) in patients without EMG alteration. No patient had prior vocal fold medialization therapy.

The control group consisted of 11 normal volunteers, six women and five men aged 29 - 72 years, median 58 years. They did not have dysphagia, neurological diseases, heartburn, or acid regurgitation, nor were they receiving treatment for any disease. They were recruited by advertisement inside the hospital. The study was approved by the Human Research Ethics Committee of the University Hospital of Ribeirao Preto. Written informed consents were given by all patients and volunteers.

The videofluoroscopic examination was performed with an Arcomax Phillips model BV 300 instrument (Veenpluis, The Netherlands), and with the digital image processing system Ever Focus model EDSR 100 V 1.2 (Taipei, Taiwan) with a DVR monitor (Ever Focus) running at 60 frames/second, and a clock time that indicates digital time in hundredths of a second on each video frame.

Each subject was studied while sitting in a chair, turning laterally to the image intensifier. Lateral images were obtained from the mouth, pharynx, and proximal esophagus. The patients swallowed in duplicate 5 and 10 mL of liquid barium (100% Bariogel, Laboratorio Cristalia, Itapira, SP, Brazil), and 5 and 10 mL of paste barium prepared with 50 mL of liquid barium plus 4.5 g of instant food thickener (Thick & Easy, Hormel Health Labs, Austin, MN, USA).

It was timed the following events: 1) onset of propulsive tongue tip movement towards the maxillary incisors; 2) onset of tongue base movement; 3) onset and end of the hyoid movement; 4) passage of the bolus head through the fauces (onset of pharyngeal phase); 5) passage of the bolus tail through the fauces; 6) onset and offset of UES opening [9]. Based on these timings, we calculated the oral transit (from tongue tip at the incisors to passage of the bolus tail through the fauces), pharyngeal transit (from bolus tail at fauces to the closure of UES), pharyngeal clearance (entry of the bolus head into the oropharynx, when it passes the fauces, until UES closure), UES transit duration (time between onset and offset of UES opening), and duration of hyoid movement (time between onset and end of the hyoid movement). Laryngeal penetration was seen when the contrast entered the airway and remained above the vocal folds, and aspiration when the contrast passed the glottis [4].

Statistical analysis was performed at the Quantitative Methods Center (CEMEQ) of the Medical School of Ribeirao Preto USP, using the Graph Pad InStat 3 software (Graph Pad Software Inc., San Diego CA, USA). The unpaired Student t-test and Fisher test were used for comparison between groups in the analysis of the timing and of the proportion of subjects with penetration and residues, respectively. The difference was considered significant when p < 0.05 in a two-tailed statistical analysis. The results are reported in seconds as mean, standard deviation, and median.

## Results

**Figure 1:**
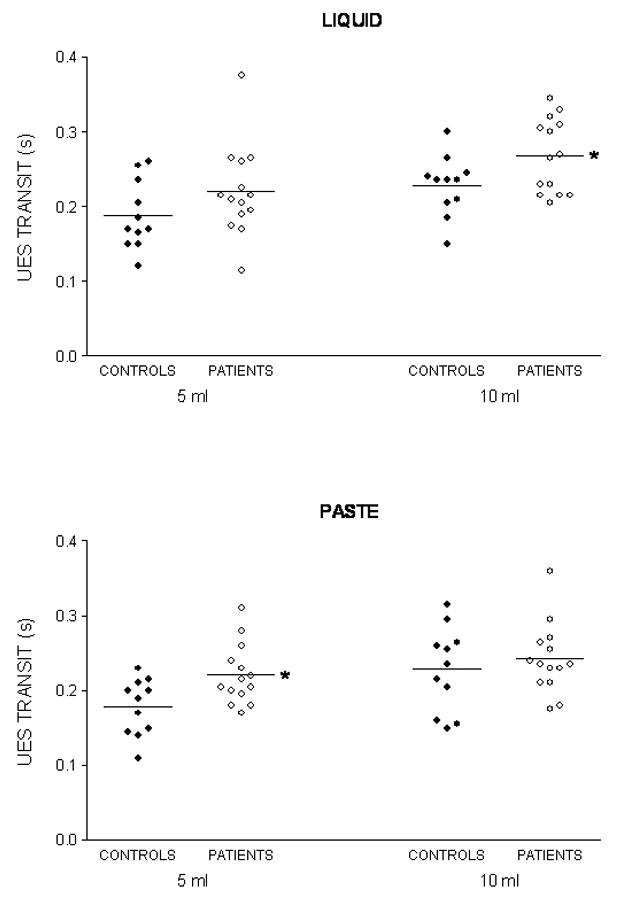
Upper esophageal sphincter (UES) transit duration after swallows of 5 mL and 10 mL of liquid and paste barium in patients with unilateral vocal fold immobility and controls. The horizontal bar represents the mean. * p < 0.04 vs controls.

The oral and pharyngeal transit duration, the UES transit duration, and hyoid movement duration are shown in Table 1 for the liquid bolus and in Table 2 for the paste bolus. The significant difference found between patients and controls was in UES transit duration, which was longer in patients with swallows of a 10 mL liquid bolus (p = 0.03) and with swallows of a 5 mL paste bolus (p = 0.01). Figure 1 shows the individual results of the UES transit duration.

**Table 1:** Oral and Pharyngeal Transit Duration in Patients With Unilateral Vocal Fold Immobility (n = 14) and Controls (n = 11) After Swallowing 5 mL and 10 mL Liquid Bolus (Seconds)

	5 mL				10 mL			
	Controls		Patients		Controls		Patients	
	Mean ± SD	Median	Mean ± SD	Median	Mean ± SD	Median	Mean ± SD	Median
**Oral transit**	0.69 ± 0.37	0.57	0.49 ± 0.39	0.37	0.33 ± 0.10	0.31	0.51 ± 0.47	0.36
**Pharyngeal Transit**	0.19 ± 0.06	0.20	0.20 ± 0.05	0.20	0.22 ± 0.16	0.18	0.21 ± 0.05	0.21
**Pharyngeal clearance**	0.45 ± 0.19	0.42	0.46 ± 0.38	0.34	0.36 ± 0.09	0.39	0.49 ± 0.31	0.36
**UES transit**	0.19 ± 0.05	0.18	0.22 ± 0.06	0.21	0.23 ± 0.04*	0.24	0.27 ± 0.05	0.27
**Hyoid movement**	0.60 ± 0.26	0.47	0.59 ± 0.28	0.50	0.53 ± 0.24	0.42	0.65 ± 0.38	0.47

* p = 0.03 vs patients

**Table 2:** Oral and Pharyngeal Transit Duration in Patients With Unilateral Vocal Fold Immobility (n = 14) and Controls (n = 11) After Swallowing 5 mL and 10 mL Paste Bolus (Seconds)

	5 mL				10 mL			
	Controls		Patients		Controls		Patients	
	Mean ± SD	Median	Mean ± SD	Median	Mean ± SD	Median	Mean ± SD	Median
**Oral transit**	0.61 ± 0.45	0.49	0.60 ± 0.44	0.46	0.39 ± 0.18	0.36	0.70 ± 0.64	0.52
**Pharyngeal Transit**	0.20 ± 0.08	0.20	0.29 ± 0.22	0.22	0.20 ± 0.06	0.22	0.23 ± 0.05	0.24
**Pharyngeal clearance**	0.54 ± 0.32	0.58	0.44 ± 0.32	0.34	0.63 ± 0.42	0.50	0.62 ± 0.54	0.35
**UES transit**	0.18 ± 0.04*	0.20	0.22 ± 0.04	0.21	0.23 ± 0.06	0.24	0.24 ± 0.05	0.24
**Hyoid movement**	0.57 ± 0.22	0.48	0.55 ± 0.22	0.45	0.68 ± 0.26	0.70	0.56 ± 0.21	0.50

* p = 0.01 vs patients

**Figure 2:**
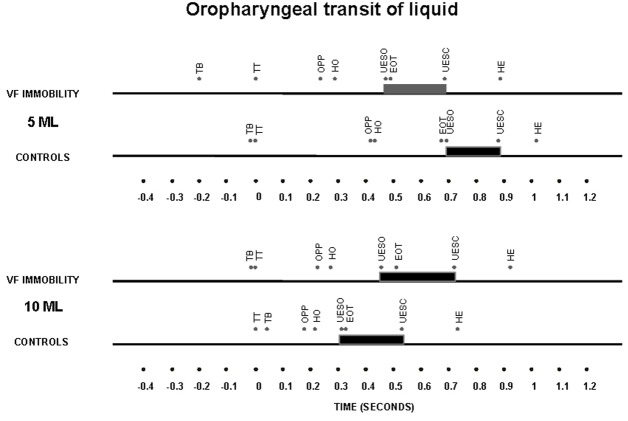
Timing represented by the means of oral and pharyngeal transit after swallows of a 5 mL and 10 mL liquid bolus in patients with unilateral vocal fold immobility and controls. TT – tongue tip at the maxillary incisors; TB – onset of tongue base movement; OPP – onset of pharyngeal phase, bolus head into the oropharynx; HO – onset of the hyoid movement; EOT – end of oral transit; UESO – onset of the upper esophageal sphincter opening; UESC – end of upper esophageal sphincter opening; HE – end of the hyoid movement. The horizontal thick bar represents the UES transit.

**Figure 3:**
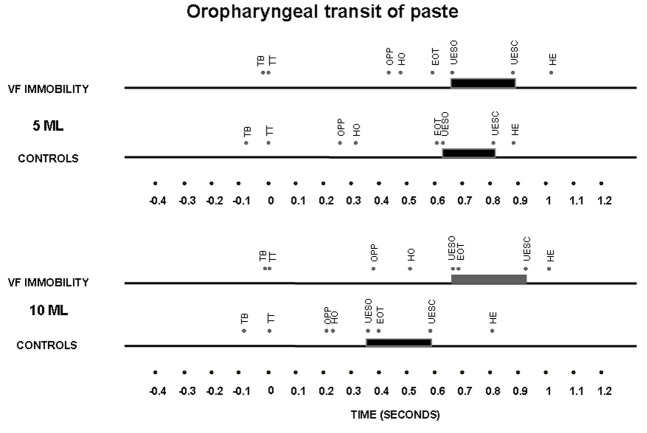
Timing represented by the means of oral and pharyngeal transit after swallows of 5 mL and 10 mL of a paste bolus in patients with unilateral vocal fold immobility and controls. TT – tongue tip at the maxillary incisors; TB – onset of tongue base movement; OPP – onset of pharyngeal phase, bolus head into the oropharynx; HO – onset of the hyoid movement; EOT – end of oral transit; UESO – onset of the upper esophageal sphincter opening; UESC – end of upper esophageal sphincter opening; HE – end of the hyoid movement. The horizontal thick bar represents the UES transit.

The timing of swallow-related events, indexed to propulsive tongue tip movement at the maxillary incisors as the zero reference for liquid bolus (Fig. 2) and for paste bolus (Fig. 3), showed that the time of UES opening in the control subjects had a temporal dependence on bolus volume for both liquid and paste barium, with an early opening when the bolus volume increase from 5 mL to 10 mL, an effect that was not seen in patients with unilateral vocal fold immobility (Table 3).

**Table 3:** Timing of Onset of Hyoid Movement and Onset of Upper Esophageal Sphincter (UES) Opening, Indexed to Propulsive Tongue Tip Movement at the Maxillary Incisors as the Zero Reference, After Swallowing a 5 mL and 10 mL Liquid and Paste Bolus in Patients With Vocal Fold Immobility and Controls (Seconds)

	Controls				Patients			
	5 mL		10 mL		5 mL		10 mL	
	Mean ± SD	Median	Mean ± SD	Median	Mean ± SD	Median	Mean ± SD	Median
**Liquid**								
Hyoid movement	0.43 ± 0.36	0.35	0.22 ± 0.24	0.17	0.29 ± 0.40	0.19	0.28 ± 0.45	0.23
UES opening	0.69 ± 0.35*	0.51	0.32 ± 0.20	0.29	0.47 ± 0.39	0.35	0.45 ± 0.45	0.32
**Paste**								
Hyoid movement	0.31 ± 0.51	0.25	0.25 ± 0.22	0.20	0.47 ± 0.56	0.38	0.50 ± 0.58	0.32
UES opening	0.63 ± 0.42**	0.50	0.37 ± 0.21	0.35	0.67 ± 0.55	0.56	0.69 ± 0.64	0.53

*p = 0.003 vs 10 mL ** p = 0.049 vs 10 mL

There was no difference between patients with or without alteration of EMG activity (Table 4). No patients or controls had aspiration during the swallowing evaluation. Laryngeal penetration was seen with a liquid bolus in 21% of patients with unilateral vocal fold immobility and in 9% of control subjects. In 27% of control subjects and in 43% of patients with vocal fold immobility, there were pharyngeal residues, more frequently seen with the liquid bolus in controls (80%) and with the paste bolus in patients (83%). With the paste bolus, all patients with abnormal EMG and 67% of patients with normal EMG had residues.

**Table 4:** Pharyngeal transit duration and upper esophageal sphincter (UES) transit duration in patients with unilateral vocal fold immobility, with normal electromyographic (EMG) evaluation (n = 6) and with abnormal EMG evaluation (n = 8), after swallows of 5 mL and 10 mL of a liquid and paste bolus (seconds)

	Pharyngeal Transit				UES Transit			
	Normal EMG		Abnormal EMG		Normal EMG		Abnormal EMG	
	Mean ± SD	Median	Mean ± SD	Median	Mean ± SD	Median	Mean ± SD	Median
**Liquid**								
5 mL	0.19 ± 0.06	0.17	0.21 ± 0.05	0.24	0.22 ± 0.08	0.22	0.22 ± 0.04	0.21
10 mL	0.20 ± 0.04	0.19	0.22 ± 0.06	0.22	0.27 ± 0.05	0.25	0.27 ± 0.05	0.28
**Paste**								
5 mL	0.35 ± 0.34	0.22	0.24 ± 0.05	0.21	0.21 ± 0.03	0.21	0.23 ± 0.05	0.21
10 mL	0.21 ± 0.04	0.22	0.25 ± 0.06	0.24	0.22 ± 0.04	0.23	0.26 ± 0.05	0.25

## Discussion

It was found in patients with unilateral vocal fold immobility a longer transit of liquid and paste barium through the UES compared with normal volunteers and no adaptation of the timing of UES opening to the increase in the bolus volume swallowed.

The UES is under high pressure most of the time but with frequent increases and decreases caused by physiologic stimuli, and intermittently opening and closing to allow passage of contents during physiologic events [10]. The most important component of the UES is the cricopharyngeal muscle, which receives innervation from the pharyngeal plexus which in turn is supplied by the pharyngeal branch of the vagus nerves (pharyngoesophageal nerve), superior laryngeal nerve, and recurrent laryngeal nerve, and also by the glossopharyngeal nerve and sympathetic nerve fibers from the superior cervical ganglion [10], with the recurrent laryngeal nerve providing no motor innervation [10, 11].

Normal UES opening and transit involve sphincter muscle relaxation, anterior laryngeal traction, and intrabolus pressure [12]. Superior and anterior excursion of the larynx during swallowing opens the UES and enlarges the pharynx to receive the swallowed bolus. The pressure force generated by the swallowed bolus also contributes to UES opening [12]. Limitations in the capacity of the pharynx to generate pressure and in laryngeal excursion affect UES transit. Multiple abnormalities of pharyngeal function were identified in patients with paresis of the recurrent nerve, such as defective closure of the laryngeal vestibule, defective apposition of the corniculate cartilages, defective opposition of the arytenoid cartilages, defective movement of the epiglottis, and paresis of the pharyngeal constrictor musculature when there was also paresis of the superior laryngeal nerve [6]. Some patients with vocal fold immobility had abnormal pharyngeal stripping wave and pharyngeal retention [13], but UES opening impairment was not found in these patients [1, 13]. The longer UES transit should be the consequence of the impossibility of the pharynx to generate enough pressure [13], longer pharyngeal contraction duration [7], and a decrease in the subglottic air pressure [14]. Positive subglottic pressure during swallowing is required for an efficient swallow. Subglottic pressure decreases the distensible volume of the swallow and avoids dissipation of the intrabolus pressure occurring during swallows [15]. These situations, inadequate pharyngeal pressure, decrease in the subglottic air pressure, and longer pharyngeal contraction duration, should be the explanation for the longer UES transit for patients with pharyngeal innervation impairment and patients without clear innervation impairment. Patients whose vocal fold immobility is of idiopathic etiology have similar alterations in pharynx and UES to those shown by patients with recurrent pharyngeal nerve impairment [7]. The slower UES transit, and consequent longer UES opening duration, may cause dysphagia and increase the number of subjects with pharyngeal residues.

The absence of adaptation of UES opening timing to the increase in bolus volume in the patients also suggests the impairment of pharyngeal function, which may also be related to the increase in laryngeal penetration and aspiration [6], and possibly reflects changes in pharyngeal sensitivity or in the control of pharyngeal motor activity. In normal subjects, increases in bolus volume do not cause alteration in pharyngeal transit or clearance, but cause an early onset of anterior laryngeal movement and an early UES opening [9, 16, 17]. UES relaxation and contraction responses are triggered by stimulation of sensory afferents proximally from the pharynx and distally from the esophagus. These responses are mediated by the brain stem and have as efferent targets the component muscles of the UES and selected muscle groups that exert a distracting force on the hyoid bone [18].

The results confirm previous reports showing that unilateral vocal fold immobility is most commonly left-sided [1, 19], and that penetration is more frequent with a liquid bolus than with a paste bolus [4]. Laryngeal penetration was described in 31.3% of the patients and laryngeal aspiration in 23.4% [1]. Among patients evaluated for dysphagia who had vocal fold immobility, laryngeal penetration was seen in 53% with a liquid bolus and in 47% with a puree bolus consistency, with aspiration present in 44% of patients [19]. The patients included in this investigation had no aspiration but 21% of them had laryngeal penetration with a liquid bolus. The longer interval between the occurrence of vocal fold immobility and the swallowing evaluation, compared with previous data [19], may be the explanation for the observation of less frequent penetration and aspiration.

The increased frequency of patients with pharyngeal residues also raises the possibility of penetration and aspiration. There is a significant association between the presence of bolus residue and a higher penetration-aspiration scale score, which highlights bolus residue as a risk factor for postswallow aspiration in patients with vocal fold immobility [1].

There was no difference between patients with abnormal and normal EMG examination. It is possible that the number of patients in each group was not large enough to show significant differences. The time between the voice alteration caused by vocal fold immobility and the swallowing evaluation, longer in patients with normal EMG than in patients with abnormal EMG, may have affected the results. A previous study on 34 patients with vocal fold immobility showed a normal EMG pattern in the thyroarytenoid and cricothyroid muscles of 6 patients (17.6%), a neuropathic pattern in 26 (76.5%), and myopathy in two (5.9%) [1]. In this paper, 8 (57.1%) had alteration of the EMG pattern and 6 (42.9%) did not. The specificity of EMG has been described as 100% in detecting vocal fold immobility, with 65.7% sensitivity. In predicting recovery, the specificity of EMG is 100% and the sensitivity is 86.6% [20]. The six patients with normal EMG examination might have had vocal fold fixation rather than neural paralysis. These patients often exhibit a degree of glottic insufficiency similar to that of patients with vocal cord immobility caused by neural paralysis [1], causing a persistently open airway, which impairs the necessary negative hypopharyngeal pressure needed to propel the bolus through the pharynx [1, 15]. It is possible that some of the patients with normal EMG examination had false-negative results, because the sensitivity of the method is not perfect [20].

In conclusion, the results show that during swallowing, when compared with normal subjects, patients with unilateral vocal fold immobility may have a longer UES transit duration and no early UES opening when bolus volume increases.
